# Frequency Domain Mapping of Atrial Fibrillation - Methodology, Experimental Data and Clinical Implications

**DOI:** 10.2174/157340312803217229

**Published:** 2012-08

**Authors:** Vassil B Traykov, Róbert Pap, László Sághy

**Affiliations:** * Department of Electrophysiology, Clinic of Cardiology and Angiology, Tokuda Hospital, Sofia, Bulgaria; § Second Department of Internal Medicine and Cardiology Centre, University of Szeged, Hungary

**Keywords:** Atrial fibrillation, frequency domain analysis, dominant frequency, fast Fourier transform.

## Abstract

The concept of dominant frequency (DF) has been used as a way to express local atrial activation rate during atrial fibrillation (AF). The rotor theory explaining the pathophysiology of AF is widely based upon spatial distribution of DF in the atria. Using frequency domain analysis to represent the rate of atrial activation by DF can avoid some of the limitations of time domain analysis of signals during AF. Understanding the concept of DF is of utmost importance to the proper use and interpretation of frequency domain analysis in AF. The current review focuses on the basic principles and methodology of frequency domain analysis using the Fourier transform during different types of AF. It also provides an update of the published experimental and clinical data on frequency domain analysis in light of the rotor theory for AF maintenance.

## INTRODUCTION

Atrial fibrillation (AF) is the most common supraventricular arrhythmia in humans. For many years it has been considered a "random" process. However, research during the last decade has indisputably shown that there is a pattern of organization in this arrhythmia. The landmark discovery of Haissaguere *et al*. that AF is frequently initiated by ectopic beats arising from the pulmonary veins (PVs) has shed light on its initiation [1] but the mechanisms of AF maintenance are still a subject of debate. Moe *et al*. first attempted to explain AF maintenance by the multiple wavelet hypothesis [2] that was later corroborated by the studies of Allesie *et al* [3]. In recent years there is cumulating evidence from animal experiments and human data that AF is maintained by rotors that demonstrate fast regular activity and fibrillatory conduction to the rest of the atria producing the well known irregularly irregular ECG pattern of AF [4 -6]. This theory fuelled research into accurate mapping and representation of atrial rate throughout the atria during AF because sites of high atrial rate might represent structures critical to AF maintenance and amenable to ablation. One possible way to achieve this is by measuring and averaging the cycle length (CL) of atrial activation at different sites across the atria in the time domain [7,8]. Fragmented and irregular atrial electrograms frequently present a major obstacle to accurate CL measurement. Therefore another approach that is gaining popularity is to use spectral analysis of atrial electrograms and represent the local activation rate in the frequency domain [4-6, 9-12].

The current review focuses on the methodology of frequency mapping of atrial fibrillation and provides a brief review of the most important animal experiments and human studies.

## CONCEPT OF FREQUENCY DOMAIN ANALYSIS

Undoubtedly, the most widely accepted way of presenting signals in medicine and technology is the time domain where values of certain parameters (voltage, current, pressure etc.) are plotted as a function of time. With this modality what one actually sees and interprets is the temporal change in the measured parameter e.g. voltage amplitude in the ECG or intracardiac electrograms. In this case the time interval between QRS complexes on an ECG lead or between two deflections on the intracardiac electrogram will be the CL of activation of the structure the signal is recorded from (the ventricles in case of QRS CL). Periodical signals can also be presented in the frequency domain by showing the distribution and strength of different frequencies in a given time interval. There is a frequency band of highest strength that is called dominant frequency (DF). It correlates to the CL derived from the time domain analysis. So frequency domain analysis might be considered to some extent as another way to express rate of activation by using frequency and not CL. In regular tachycardias this approach is of no significant value because time domain measurements are an easy and accurate way to represent rate of activation. However, time domain measurements might be difficult and inaccurate in arrhythmias that show some irregularity in CL or signal amplitude. This is especially important when using automated algorithms to measure CL. In signals with changing amplitude some of the deflections might be undersensed and therefore not counted for CL calculation. As this is frequently the case in AF, frequency domain analysis can be very helpful in presenting atrial rate in different atrial structures. 

Frequency domain analysis is based on a series of mathematical formulas that comprise the Fourier transform. Its basic principle postulates that each continuous signal can be presented as a sum of weighted sinusoids with different frequency, amplitude and phase. The more complex the signal the more sinusoids it would take to represent it. Among those sinusoids there is one of highest amplitude whose frequency is the DF of that signal. The relative amplitude of all the sinusoids in comparison to each other is frequently termed power. Plotting values of frequencies of all the sinusoids a signal is composed of on the abscissa against their power on the ordinate produces the frequency spectrum of this signal. The peak with the highest power on this frequency spectrum represents the DF. In a perfectly regular signal the frequency spectrum would consist of one narrow DF peak equalling the inverse value of the signal CL and some other lower power peaks. Their frequencies are multiples of DF and are called harmonics. A very useful analogy which can be used to help conceive the idea of frequency domain analysis is an orchestra playing. The time domain presentation is actually the music played by it but the frequency domain would represent a "snapshot" of the loudness at which different instruments are playing during a certain time point or interval. At any time point or interval some of the instruments will sound louder, therefore some frequency bands will be higher in power while the other frequency bands will be of lower power for that particular time point or interval. 

All of the above considerations are only applicable to a continuous analog signal. However, most current electrophysiology computerized systems used in practice are digital i.e they record the signal by sampling discrete values of amplitude at discrete time points during recording. This is done at some sampling rate which in most systems is usually 1000 times per second (i.e. 1000 Hz). The higher the sampling rate, the more the signal approximates the analog continuous signal. Frequency domain analysis of digital signals is carried out using discrete Fourier transform. It is a mathematical operation that converts a discrete-time signal to a discrete-frequency spectrum. A very efficient and widely used algorithm to calculate discrete Fourier transform is the Fast Fourier Transform algorithm (FFT). Signal lengths that can be analyzed by FFT are predetermined by the sampling rate such that *signal length = n/sampling rate* where n is equal to a power of 2. The maximum frequency that can be accurately detected by the FFT is limited by the Nyquist limit at half the sampling frequency above which aliasing occurs. With most systems using a sampling frequency of 1000 Hz the maximal frequency that can be included in the frequency spectrum is 500 Hz which is far above the physiological frequency range (3-20 Hz). An important parameter of FFT is frequency resolution which is equal to the sampling rate divided by the number of points sampled during recording. At a fixed sampling rate the more points [i.e. the longer the signal] the better the frequency resolution. For instance a signal of 10 sec duration at a sampling rate of 1000 times per second (1000 Hz) would have 10000 sampling points which would be equal to a frequency resolution of 0.1 Hz. With a sampling rate of 2000 Hz the frequency resolution will be higher (0.05 Hz) for the same signal.

## METHODOLOGY AND LIMITATIONS OF FREQUENCY DOMAIN ANALYSIS

Currently frequency domain analysis is widely used to estimate atrial activation in AF in the search of zones and structures with fast and regular activity. These structures are thought to be critical for AF maintenance and when examined by frequency analysis they demonstrate high DF. As shown by previously published studies high spatial resolution DF analysis might be used to demonstrate the spatial distribution of atrial DFs across the atria. This could serve to provide insight into the mechanism of different types of atrial fibrillation as well as to guide the ablation approach. As mentioned above the more regular the signal and the more the signal resembles a sinusoid wave the closer the DF would approximate the inverse value of CL. 

**Fig. (1):**
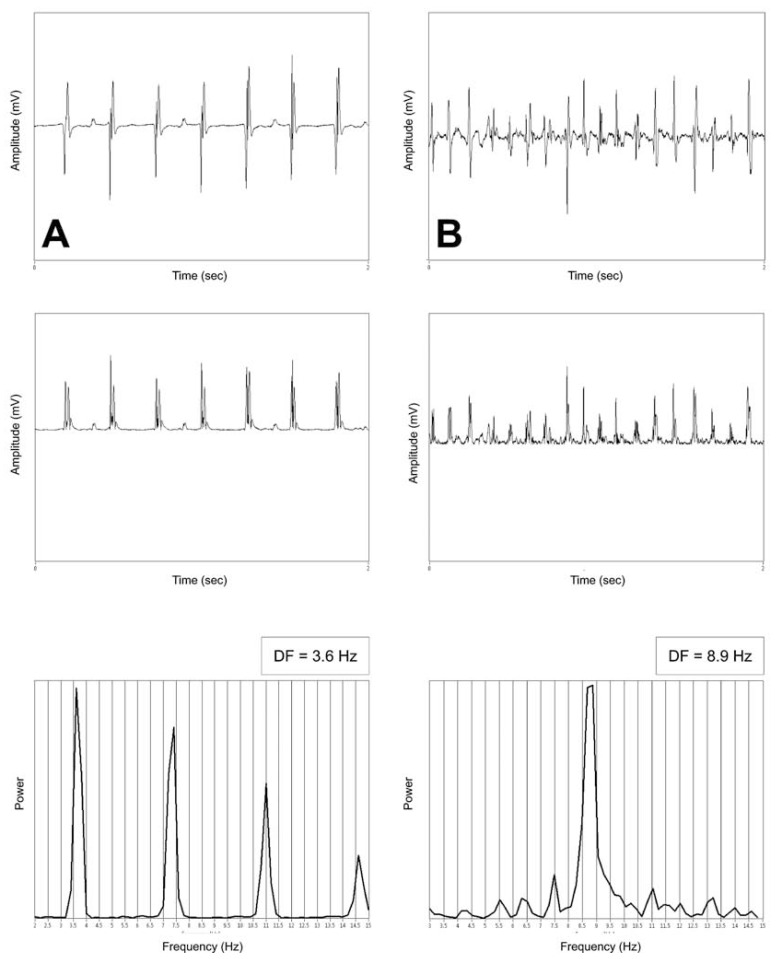
Frequency analysis in case of a regular (**A**) signal and irregular (**B**) signal with variable CL and amplitude. The upper panels represent the native signal; middle panels show the changes after rectification. The frequency spectra with the corresponding DF values are shown on the lower panels. The signal in A was recorded during typical atrial flutter with a CL of 280 msec and the signal in B is from an AF episode. Note the narrow DF peak with the harmonics in A and the wider basis of the DF peak along with the numerous smaller peaks in the frequency spectrum in B.

The signals that are usually used for FFT are those obtained during contact mapping although QRS subtracted non-contact unipolar signals have also been shown to perform well [13]. Local electrograms recorded with unipolar or bipolar electrode configurations usually consist of very sharp biphasic deflections. Decomposing those in the frequency analysis takes many sinusoids to accurately “fit” the signal. Therefore to overcome this limitation and to enhance accuracy of frequency domain analysis the signal should be subject to the following preprocessing steps agreed upon by most investigators [14,15]: 1. Bandpass filtering at 40 - 250 Hz, 2. Rectification - converts the biphasic signal into monophasic by calculating the absolute value of all the positive and negative deflections of the electrograms, 3. Windowing - attenuates the signal at the beginning and at the end of the recording in order to eliminate the effect of any abrupt changes in the time domain on the frequency spectrum. Some studies include two additional steps that are low pass filtering at 20 Hz and zero padding [14]. The former usually follows rectification and removes the frequencies that fall outside the physiological range. However its significance is controversial because interpretations of the analysis are usually done when considering the band of 3 to 15 Hz so frequencies that exceed this range are by definition excluded from any analysis. Zero padding is used to achieve higher frequency resolution from signals of short duration. With it the recorded signal is “artificially” made longer. As mentioned above a longer signal will have a higher frequency resolution at the same sampling rate. Therefore a signal of 2 sec duration which would have a frequency resolution of 0.5 Hz at a sampling rate of 1000 Hz will actually have a frequency resolution of 0.1 Hz when zero padded to 10 sec. All these steps are available in the currently marketed software packages for signal analysis such as MATHLAB (Math-works, Inc, Natick, MA, USA) and LabVIEW (National Instruments, Austin, TX, USA). (Fig. **1**) illustrates some of the steps of signal preprocessing and the power spectrum of an intracardiac recording for regular and irregular signal. Analysis was carried out by custom software application based on LabVIEW developed at our institution.

The main advantage of DF analysis is that can be easily applied to estimate the local atrial activation rate in AF. This is in contrast to interval marking in time domain where signal fractionation and varying amplitudes and CL might represent an obstacle to accurate measurement especially with the commonly used automatic marking algorithms. However, DF analysis is not devoid of limitations. Many of them are common with those of time domain analysis. These limitations have been extensively studied. In two consecutive studies Ng *et al*. elucidated the value of DF analysis by using simulated electrograms and real-time recordings obtained from epicardial mapping in animals and from endocardial recordings in AF patients [16, 17]. Their results point out to several important issues. Firstly, correlation between mean, mode and median DF and the respective values of actual atrial activation rate is only moderate to good when both amplitude and CL of the signal vary despite the length of the recording. In contrast when amplitude varies with a constant rate the correlation is close to perfect. These results are reproducible both with simulated and clinically obtained signals. Fischer *et al*. have also shown that beat to beat CL variation by more than 30% of the mean CL significantly diminishes the agreement between DF and CL [18]. Not only the magnitude of variation but also the sequence by which these changes occur can also greatly affect DF values, suggesting the role of phasic changes for the overall result. 

Averaging the values from several consecutive measurements significantly improves reproducibility and reduces the difference between DF and actual activation rate as shown by Ng *et al* in the experiments using simulated signals [16]. In contrast to that, results from animal studies report perfect correspondence between DF and CL of atrial electrograms during AF [19]. Despite averaging, signal duration also affects the correlation between DF and activation rate in the time domain. Minimal signal duration that provides good correlation has been reported by some groups to be 2 seconds [17] and 5 seconds by others [20], probably reflecting differences in study protocols. Signal duration has a great impact on time and frequency resolutions of frequency domain analysis. Longer signals will not be able to reflect dynamic changes occurring during the recording period. On the other hand, a shorter signal would have a good time resolution but will not be sensitive to small frequency changes. Therefore selecting the optimal signal duration when performing frequency domain analysis is always a result of a compromise between time resolution and frequency resolution. Signals recorded from some atrial regions i.e. the distal portion of the coronary sinus can demonstrate far-field ventricular signals of different amplitude. According to published data they significantly affect DF values not with their rate but rather with their timing. With regular signals DF is affected when the ventricular-to-atrial signal ratio is equal or more than one while with irregular signals (such as those in AF) DF values are affected when this ratio is as low as 0.5. Given the low amplitude of atrial signals that are usually recorded during AF this ratio appears to be quite restrictive. Therefore, signals that contain significant far field ventricular potentials should be either excluded from the analysis [10] or subject to offline ventricular template subtraction during signal preprocessing. The latter has been shown to reduce differences between frequency and time-domain parameters [17]. With relatively large-amplitude signals the presence of far-field ventricular electrograms results in falsely higher DF. In those cases a harmonic is usually taken as the DF. When atrial signal amplitudes are lower DF might also be falsely lower due to DF being calculated at a harmonic of DF of the ventricular rather than the atrial signal. Analysis of signals with widely spaced double potentials can lead to "double counting" and taking a harmonic for the DF peak in the spectrum even with perfectly regular signals making time domain analysis more appropriate in this situation [16]. Noise is another source of discrepancies between DF and actual atrial rate more so in signals with varying amplitude and rate. Signal-to-noise ratio below 13 dB could also affect DF values especially for signals demonstrating large variations in timing and/or amplitude [17].

**Fig. (2):**
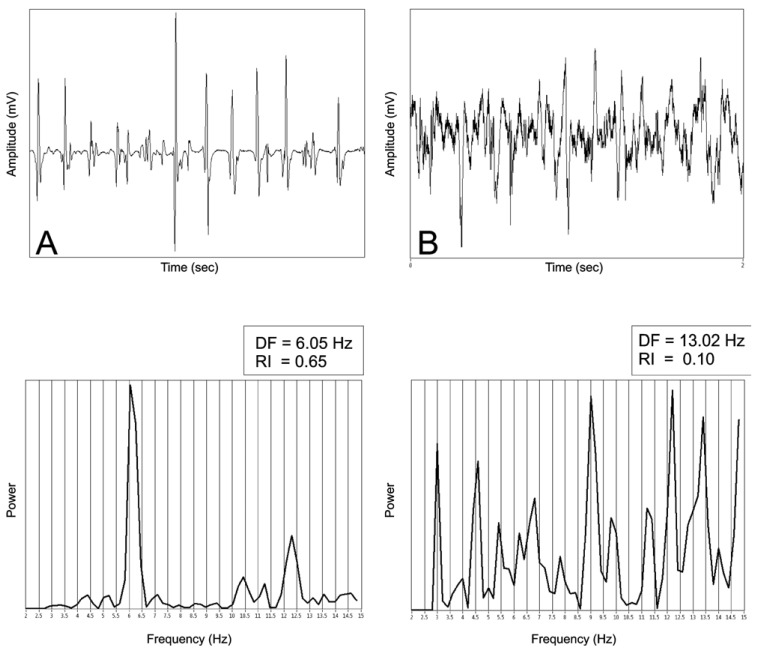
Frequency analysis of a signal with slight degree of fragmentation and small CL variation (**A**) and of a signal with very fragmented electrograms and significant CL variation (**B**). The native signals in both cases are shown in the upper panels and lower panels demonstrate the frequency spectra of the signals with the corresponding values of DF and RI. Note that in B the frequency spectrum has numerous additional high power peaks apart from the DF peak making accurate interpretation questionable. Both signals were recorded during AF.

Power spectrum can also provide reliable information about regularity of the signal. The more irregular and fractionated a signal is the more additional peaks it would have on the power spectrum apart from the DF and its harmonics because more sinusoids with different frequencies would be needed to “fit” the signal. Calculating the area under the DF peak and its harmonics and presenting it as a ratio to the total area under the power curve has been termed regularity index (RI) and has been successfully used as a measure of regularity [9, 21]. RI can serve two purposes. Firstly, it can aid in recognizing AF drivers which demonstrate fast and regular activity as discussed in detail below. Secondly, it can render DF analysis more reliable by excluding recordings with low RI (usually below 0,2) thus avoiding the inaccuracies associated with very irregular and fractionated signal. An example of signals with high and low RI is shown on (Fig. **2**).

## IMPLICATIONS OF DF ANALYSIS IN AF

After the era of multiple wavelet hypothesis much research has focused on identifying drivers and demonstrating organized patterns during AF. A number of studies mainly performed during open heart surgery have demonstrated some degree of organization in some parts of the atria activated with a shorter CL, usually in the left atrium (LA) but also in the right atrium (RA) [7, 22-29]. This data along with the advent of optical mapping set the path for further studies on spatial and temporal periodicity of AF. Most of these studies used FFT to assess frequency distribution in the atria during AF with the concept that a driver with regular activation maintaining AF would demonstrate a single narrow peak on the frequency spectrum. In a canine model of pacing induced congestive heart failure Ryu *et al*. showed with FFT that the most common pattern of activation during AF demonstrated biatrial drivers with a LA-to-RA frequency gradient [30]. Another study using optical and contact mapping during AF in sheep hearts has elegantly shown the presence of spatiotemporal periodicity with driver regions termed rotors, most commonly located in the LA and demonstrating a very short CL of activation. Their CL correlated strongly with the DF in that zone and was also represented as a peak in the biatrial frequency spectrum. Optical mapping suggested reentry at least in some of these rotors [4]. The same group went further and demonstrated that these reentrant rotors driving AF in the isolated sheep heart are temporally stable and predominantly localized in the pulmonary venous ostia and LA posterior wall [5]. The rest of the atria cannot be activated in 1:1 fashion at that short CL which results in wave breakdown and development of complex block patterns especially in the RA pectinate musculature termed fibrillatory conduction. Wave breakdown occurring above a certain frequency (respectively below a certain CL) was reflected on the spatial distribution of DF by the formation of multiple well-demarcated DF domains throughout both atria - a landmark of fibrillatory conduction [6]. As demonstrated by Mansour *et al*. these DF domains are spatially distributed in a hierarchical order with the highest DF located in the LA and the lowest in the RA thus giving rise to a LA-to-RA frequency gradient [9]. The same authors also showed that the gradient strongly correlates with the maximal DF and it might increase after severing major interatrial connections due to a drop in RA maximal DF without change in LA maximal DF. This suggests a major role for the rotors as drivers of AF and makes multiple wavelets an unlikely mechanism of AF maintenance in this setting. Other authors have also shown the presence of LA to RA frequency gradient in canine models of both acute and chronic AF although in the latter case frequencies were higher in both LA and RA [31]. The frequency of AF drivers has been shown to increase with increasing atrial stretch caused by elevated intracavitary pressure in the LA in the experimental setting [32]. 

**Fig. (3):**
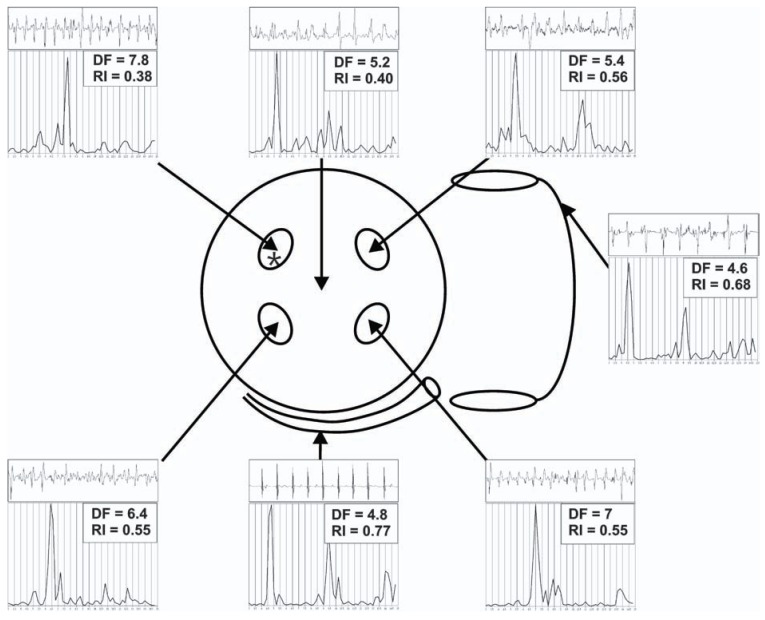
Frequency analysis demonstrating spatial distribution of DFs derived from signals recorded from the four PV ostia, LA posterior wall, RA/superior vena cava junction and the coronary sinus in a patient with paroxysmal AF. The frequency spectra of the recorded signals are also presented. Note that the highest DF was recorded at the ostium of the left superior PV which was identified as arrhythmogenic in this patient as denoted by the asterisk. Another high DF site was recorded at the ostium of the right inferior PV. Note the lower DF at the coronary sinus and the lowest DF at the RA/superior vena cava junction demonstrating a significant LA-to-RA gradient. The atria are schematically represented as viewed from posterior.

FFT and DF analysis has also been widely used in human studies. Sahadevan used FFT in a surgical study to analyse epicardial atrial activation patterns in patients with chronic AF undergoing cardiac surgery [33]. Lazar *et al*. analysed DF distribution in patients with paroxysmal and persistent AF by consecutively recording electrograms from all the four PV ostia along with signals from coronary sinus and RA/superior vena cava junction [10]. Their results demonstrate the presence of a significant LA-to-RA frequency gradient in paroxysmal AF. In their series most of the sites with highest DF in paroxysmal AF patients were found to be located at the PV/LA junction suggesting that PV ostia might host drivers maintaining AF. Similar findings have been reported by other groups as well [34, 35]. Lin *et al*. studied the differences in spatial distribution in DF in patients with paroxysmal AF initiated by PV ectopy and by ectopic beats originating from the superior vena cava [36]. They have found that the gradient between LA and RA is always present but its direction depends on the location of the initiating triggers. The DF was always highest in the structure that initiated AF suggesting that arrhythmogenic structures might act both as triggers and as perpetuators of paroxysmal AF. Our group has reported similar results [37] (Fig. **3**). Another very recent and elegant study has provided evidence that triggers of paroxysmal AF located in the PV ostia are surrounded by potential arrhythmogenic substrate [38]. High DF sites were reportedly located within 15 mm from the PV ostia in most patients with 75% of those associated with arrhythmogenic PVs also showing that AF triggers and perpetuators frequently colocalize. These sites were frequently surrounded by fractionated electrograms again occupying the region within 15 mm from the PV ostia suggesting the presence of arrhythmogenic substrate in these zones. Circumferential PV isolation led to AF termination in 76% of the cases, 69% of which terminated upon ablation in the 15 mm zone surrounding the arrhythmogenic PVs.

In persistent AF, DFs are reported to be higher across the atria and some groups do not report a significant LA-to-RA or intra-LA frequency gradient [10, 39]. This might be explained by an increase in RA DF possibly due to remodelling or to the presence of an alternative mechanism for AF maintenance in these patients. A recent study using frequency mapping has provided evidence that some cases of persistent AF are likely to be maintained by a “background tachycardia” that can be detected by its DF peak on the frequency spectrum before any ablation is performed. This tachycardia has been shown to be reentrant in origin with a circuit distant from the ablation sites. This data supports the role of extra-PV substrate for AF maintenance in persistent AF cases and might serve to explain the reported lack of significant intraatrial frequency gradients in those cases [39]. Interestingly, these findings have not been corroborated by another study that shows a small but significant LA-to-RA DF gradient with centrifugal reduction in DF and regularity away from the highest DF site located in the LA in the majority of patients [40]. The centrifugal pattern of DF distribution and regularity across the atria suggests the presence of driver regions with very fast and regular activity maintaining the fibrillatory process. An intra-LA gradient was demonstrated in persistent AF in patients with significant left ventricular systolic dysfunction but not in the persistent cases with normal ventricular function [35]. In the former case the gradients were due to high DFs at the left atrial appendage which was a region spared by fibrosis in the patients with LV dysfunction. A plausible explanation for the higher mean DFs in persistent AF is provided by a very recent study examining the impact of LA pressure on DF [41]. Its results show that in persistent AF LA pressure was higher as compared to paroxysmal cases. LA pressure was shown to correlate well with DF values measured at the LA appendage supporting the role of atrial stretch for AF maintenance by atrial remodeling and stabilization of high-frequency AF drivers and providing proof for the higher mean DF in persistent AF.

PV isolation in paroxysmal AF has been shown to result in DF gradient abolition while long term outcome of catheter ablation in persistent AF was better in patients with a larger baseline DF gradient [11]. A very recent study reports data further supporting the role of PVs for the maintenance of paroxysmal AF [42]. In that study termination rates of AF were higher when the PV showing the highest DF among all PVs was isolated. Interestingly the termination rate was up to 89% when PVs showing the highest DF across the atria were isolated. Sanders *et al*. have done retrospective DF analysis in a population with paroxysmal and long-standing persistent AF undergoing ablation and have integrated the DF data with a 3D map showing the colour coded distribution of DF across LA and RA [12]. Their results demonstrate the presence of a LA-to-RA gradient in both groups of patients but with significantly higher DFs across the atria in long-standing persistent AF. In paroxysmal AF sites of high DF tended to cluster mainly at PV/LA junction while in permanent cases they were more dispersed in both atria. Interestingly in 87% of the paroxysmal cases that had AF termination with PV isolation arrhythmia stopped during ablation at a high DF site. In the remaining patients maps showed that the high DF sites were not located at the PV region emphasizing the role of high DF sites for AF maintenance and the importance of identifying these sites especially in paroxysmal AF. Recently Atienza *et al*. have performed real-time DF-guided ablation using a software tool integrated in the CARTO™ three-dimensional mapping system in patients with paroxysmal and persistent AF [43]. Their results demonstrate that PV region hosts high DF sites in paroxysmal cases in contrast to patients with persistent AF in whom highest DFs were more likely to be found in the LA. Ablation at DF sites as an initial step led to AF termination in more than 50% of paroxysmal cases. After this DF-guided ablation the LA-to-RA gradient was abolished in both groups of patients. Interestingly, the presence of such a gradient at baseline was associated with a better long-term result in persistent AF and ablation at DF sites was also predictive of long term freedom from arrhythmia in both groups of patients as shown by others for persistent AF [12]. The significant decrease of DF (≥ 11 %) at the CS and in lead V1 during stepwise catheter ablation of persistent AF (including PV isolation and CFAE ablation) was shown to be a strong outcome of the procedure comparable to termination of AF during ablation [44]. 

Although demonstration of frequency gradients and their abolition with PV isolation supports the role of drivers in AF maintenance it does not provide insight into the exact mechanisms responsible for their presence in humans. Although persistent, rapidly firing foci from the pulmonary veins have been demonstrated to be present during AF [45] more recent studies have found conclusive evidence that drivers of AF behave like reentrant circuits rather than as foci at least in paroxysmal cases [46]. 

## CONCLUSION

Frequency analysis is a powerful tool to represent spatial distribution of atrial activation rate in AF where fragmented electrograms and irregular CL prevent accurate measurement in the time domain. Its applicability has been shown in many animal experiments and human studies. Using this method LA-to-RA DF gradients can be demonstrated in paroxysmal AF and at least in some series of patients with persistent AF. Termination of AF has been shown to occur frequently with ablation at high DF sites and good results from DF-guided ablation have been reported. All this data emphasizes the crucial role of high DF sites for AF maintenance making them a potential ablation target. Thus, frequency domain mapping can provide feasible additional information during AF ablation procedures and might lead to higher success rate of this procedure. However, one has to be aware of the limitations of the method and interpret DF data accordingly.
